# Physio-Biochemical Integrators and Transcriptome Analysis Reveal Nano-Elicitation Associated Response during *Dendrocalamus asper* (Schult. and Schult. F.) Backer ex K. Heyne Micropropagation

**DOI:** 10.3390/genes14091725

**Published:** 2023-08-29

**Authors:** Anita Kumari, Shubham Joshi, Aqib Iqbal Dar, Rohit Joshi

**Affiliations:** 1 Division of Biotechnology, CSIR-Institute of Himalayan Bioresource Technology, Palampur 176061, India; anitakumarii.som@gmail.com (A.K.); shubham1431996@gmail.com (S.J.);; 2 Academy of Scientific and Innovative Research (AcSIR), Ghaziabad 201002, India

**Keywords:** bamboo, gold nanoparticles, RNA sequencing, tissue culture, transcriptome, transcription factors

## Abstract

Bamboos are perennial, arborescent, monocarpic and industrially important non-timber plants. They are important for various purposes, such as carbon sequestration, biodiversity support, construction, and food and fiber production. However, traditional vegetative propagation is insufficient for bamboo multiplication. Moreover, little is known about the mechanism of gold nanoparticles (AuNPs) in vitro proliferation and regulation of physiological and biochemical properties. In this study, we investigated the impacts of citrate and cetyltrimethylammonium bromide (CTAB) coated AuNPs on in vitro proliferation, photosynthetic pigment content and antioxidant potential of *Dendrocalamus asper* (Schult. and Schult. F.) Backer ex K. Heyne. Various morpho-physiological and biochemical parameters were differentially affected along the citrate- and CTAB-coated AuNPs concentration gradients (200–600 µM). In vitro shoot proliferation, photosynthetic pigment content and antioxidant activities were higher in *D. asper* grown on Murashige and Skoog medium supplemented with 2 mg·L^−1^ benzyladenine and 400 µM citrate-coated AuNPs than in those grown on Murashige and Skoog medium supplemented with 600 µM CTAB- coated AuNPs. Identification of genes regulating in vitro *D. asper* proliferation will help understand the molecular regulation of AuNPs-mediated elicitation for modulating various physiological and biochemical activities during micropropagation. Gene Ontology enrichment analysis and Kyoto Encyclopedia of Genes and Genomes pathway analyses identified differentially expressed genes associated with in vitro modulation of AuNPs-regulated biological processes and molecular functions. The findings of this study provide new insight into AuNPs-mediated elicitation of in vitro mass scale bamboo propagation.

## 1. Introduction

*Dendrocalamus asper* (Schult. & Schult. F.) Backer ex K. Heyne, also called giant bamboo or dragon bamboo, is a massive, tropical, sympodial bamboo species indigenous to Southeast Asia. Due to its large diameter of approximately 8–12 cm and 15–20 m tall straight culms, this bamboo has been utilized as a building material in heavy construction since time immemorial. Young *D. asper* shoots are sweet in taste and sold in canned or vacuum-packed packets [1]. Bamboo shoots can also be used to extract high-quality fiber for modern textile and clothing industries [2]. However, for continuous bamboo supply, new and sustainable propagation methods with reduced proliferation period are necessary to meet industrial demands. Thidiazuron (TDZ) supplemented Murashige and Skoog (MS) medium increases shoot proliferation of *D. asper* [3]. MS media supplemented with benzyladenine (BAP; 2 mg·L^−1^), kinetin (1 mg·L^−1^) and Naphthalene acetic acid (NAA; 0.5 mg·L^−1^) optimally induces callus formation and organogenesis [4]. Gonçalves et al., 2023 [5] reported that MS media supplemented with 2–3 mg·L^−1^ BAP and 4 mg·L^−1^ indole-3-butyric acid (IBA) resulted in shoot and root growth, respectively. Moreover, researchers have used various elicitors, including nano-particles (NPs) with various physical and chemical properties, to increase in vitro calli and shoot proliferation [6,7,8,9,10,11,12].

NPs are molecules or atomic aggregates ranging in size from 1 to 100 nm [6]. The concentration, size and physical characteristics of NPs affect plants in a species-specific and developmental stage-specific manner [7]. Engineered NPs have attracted much interest because of their prospects in increasing crop productivity. The progress in dedifferentiation, genetic transformation, biomass increment, reduced infection and proliferation of advantageous secondary metabolites have all benefited through nano-biotechnology [7]. Various nano-materials have been shown to penetrate the seed coat and improve water absorption and utilization by seeds [8]. Fe/SiO_2_ nanomaterials have shown to enhance germination of barley and maize seeds [9]. Metal NPs have large surface areas and can exchange valance electrons with biomolecules more readily due to high surface area to volume ratio. Therefore, metal NPs can participate in cellular redox reactions and sugar biosynthesis pathways and change the antioxidant status of plants [10]. Gold nano-particles (AuNPs) comprise an inert core material to which surface coatings can be added, thereby functionalizing them and preventing the gold from interacting with the environment. Accumulation of AuNPs in plants can affect secondary metabolism and plant growth [11]. Emamverdian et al., 2020 [12] demonstrated that silver dioxide NPs increase bamboo plant biomass by enhancing its anti-oxidant potential under Pb stress. They further showed that 500 µM SiO_2_ NPs effectively maintain plant growth under Pb toxicity.

De novo transcriptome analysis of *Phyllostachys edulis* identified genes involved in shoot elongation [13]. Thapa et al., 2022 [14] conducted a transcriptome study of in vitro *D. hamiltonii* plants to identify differentially expressed genes (DEGs) during juvenile and aged stages. However, the crucial regulatory genes involved in in vitro nano-elicitation in *D. asper* has not been studied yet. This study attempted to unravel the transcriptional programming that underlies nano-elicitation in *D. asper* plantlets grown in vitro. Transcriptomic analyses of genes underlying growth mechanisms may provide insight into key genes involved in growth, signaling and downstream activation of the oxidative stress tolerance mechanism [15]. This study revealed novel genes and their roles in enhancing *D. asper* growth and antioxidant potential. The complex signaling pathways underpinning oxidative stress-responsive signaling in *D. asper* are a functionally relevant and sustainable genomic resource for the advancement of genetic and metabolic engineering against oxidative stress.

Here we examined the effects of citrate- and cetyltrimethylammonium bromide (CTAB)-coated AuNPs on in vitro proliferation and antioxidant activities of the commercially important bamboo species i.e., *D. asper*. The objectives of this study were to: (1) identify the optimal citrate- and CTAB-coated AuNPs (citrate-AuNPs and CTAB-AuNPs) concentrations for stimulating *D. asper* shoot proliferation in vitro; (2) investigate the changes in physiological and biochemical properties of *D. asper* during in vitro nano-elicitation; and (3) assess the differential expression of genes through transcriptional profiling.

## 2. Materials and Methods

### 2.1. Plant Material, Treatment Conditions and Morphological Analysis

Aseptic in vitro cultures of *D. asper* were started from nodal explants in MS media containing BAP (2 mg·L^−1^) as described in previous study [5]. Previously synthesized and characterized citrate-AuNPs and CTAB-AuNPs were used in the present study [15]. MS media containing 3% sucrose, BAP and either citrate-AuNPs or CTAB-AuNPs at various concentrations, i.e., 200 µM, 400 µM and 600 µM, were prepared; pH was adjusted to 5.75–5.8 by adding either 1 M NaOH or HCl. Thereafter, 0.8% agar was added to the media. Nanoparticles were added post autoclaving, and Nps free MS medium supplemented with BAP was used as control. From one-month-old proliferated shoots, 8–10 culms were excised and inoculated in MS media containing different concentrations of the NPs using sterile forceps in a laminar air flow chamber. Each treatment was performed in triplicate. The cultures were incubated at 25 ± 2 °C under photoperiod of 16/8 h, light/dark for 30 days at a photosynthetic photon flux density of 50 mmol·m^2^·s^1^ using white fluorescent LED tubes (20 W; Bajaj, Pune, India) and under 55–60% of relative humidity.

After 30 days, AuNPs treated *D. asper* micro-plantlets were picturized and shoot length was recorded. For each replicate, the number of shoots and leaves, as well as the overall plant biomass were recorded. The aggregate of plantlets from each treatment were collected, cleaned with sterile deionized water and dried among filter papers to calculate fresh weight (FW). Each replicate of control and treated cultures was oven-dried at 40 °C to determine the dry weight (DW).

### 2.2. Estimation of Total Chlorophyll, Soluble Sugar, Malondialdehyde Content, Phenolics Contents and Antioxidant Enzyme Assay

Chlorophyll a, chlorophyll b, total chlorophyll and total carotenoid contents were assessed, as previously described [16]. To quantify the photosynthetic pigment contents, absorbance was assessed with a UV-visible spectrophotometer at 663, 645 and 480 nm [15]. Total soluble sugar content was estimated using the anthrone method [17]. For each sample, total soluble sugar concentration was calculated using a glucose standard curve at 620 nm (y = 0.0376x + 0.0025, R^2^ = 0.9987). Malondialdehyde (MDA) content was determined using the Heath and Packer, 1968 method [18]. Using a molar extinction coefficient of 155 mM^−1^·cm^−1^, the MDA concentration was determined from absorbance at 532 nm. Total phenolics were isolated as defined earlier [19] and expressed as mg·g^−1^ of the entire extract using the gallic acid standard curve (y = 0.0018x + 0.0048, R^2^ = 0.9986). Further, AuNPs treated plantlets and their controls were homogenized in liquid nitrogen to measure superoxide dismutase (SOD) enzyme activity. The SOD enzymatic potential was determined by measuring absorbance at 560 nm, and 50% reduction in absorbance compared to that in the control was defined as one unit of enzyme activity [20].

### 2.3. RNA Isolation, Library Construction, Illumina Sequencing, Transcriptome Assembly and Annotation

Shoots grown in vitro on MS media containing 400 µM of citrate-AuNPs or 600 µM CTAB-AuNPs were used for further transcriptome analysis. Total RNA was isolated in triplicate using iRIS solution (CSIR-IHBT, Palampur, India) [21]. Spectrophotometric analysis and polyadenylate mRNA enrichment were performed, followed by cDNA library preparation, purified cDNA library quantification and paired end library preparation, as described earlier [14]. Paired end raw reads from Illumina Nova seq were pre-processed using the quality filtering tool, NGSQC tool kit v2.3 (https://bioinformaticshome.com/tools/rna-seq/descriptions/NGS_QC_Toolkit.html#gsc.tab=0; accessed on 15 November 2022) [22]. Base quality check of raw data was performed using FastQC (version 0.11.9; https://www.bioinformatics.babraham.ac.uk/projects/fastqc/; accessed on 30 November 2022). Illumina Adapter (AGATCGGAAGAGC) was removed using trimgalore (version 0.6.7; https://software.cqls.oregonstate.edu/updates/trim_galore-0.6.7/; accessed on 13 February 2023). HISAT2 v 2.4.0 (http://daehwankimlab.github.io/hisat2/download/; accessed on 18 March 2023) was used to assemble the reads from the samples. Mapped reads were assembled using Cufflinks (version 2.2.1; http://cole-trapnell-lab.github.io/cufflinks/; accessed on 25 April 2023) and the resulting transcripts were merged using Cuffmerge (http://cole-trapnell-lab.github.io/cufflinks/cuffmerge/; accessed on 3 May 2023) for use as reference to compare DEGs.

### 2.4. Differential Expression Analysis

After removing adapter sequences and low-quality reads, BAC (control), BAT1 (400 µM citrate-treated) and BAT2 (600 µM CTAB-treated) specific filter reads were mapped and assembled with reference to *D. latiflorus* genome, and the transcript abundance was estimated and normalized to fragments per kilobase of transcript per million mapped fragments (FPKM) using the RNA-Seq by expectation-maximization (RSEM) tool [23]. EdgeR tool was used to detect differential expression of transcripts in treated plants compared with control plants, with a log fold change of 4.

The high-throughput sequencing data obtained through Cufflinks was screened for differentially expressed transcripts using the Cuffdiff tool. Significant DEGs were identified from the data; upregulated genes were defined by a log2FC ≥ 4 and corrected *p*-value ≤ 0.05, while down-regulated genes were defined by a log2F C ≤ 4 and corrected *p*-value ≤ 0.05 (q-value). Identification of significant DEGs is more statistically stringent using reference-based assembly (*D. latiflorus*). CummeRbund was used for plotting. Annotation of the hits was performed using Uniprot database, Gene Ontology (GO) and Kyoto Encyclopedia Genes and Genomes (KEGG). MeV package v.4.9.0 was used to create heatmaps of DE transcripts based on FPKM values.

### 2.5. Functional Annotation and Pathway Analysis

Differentially regulated transcription factors in treated plants were predicted using the plant transcription factor database (PlantTFDB). Venn diagrams were plotted to interpret commonly and uniquely expressed genes among control; 400 µM Citrate- and 600 µM CTAB-treated plants. GO analysis of differentially expressed genes was performed using BLAST2Go. REduce and VIsualize Gene Ontology (REVIGO) (http://revigo.irb.hr/ (accessed on 16 June 2023) visualization tool was used to summarize GO terms based on their semantic resemblances. Pathway analysis was performed by mapping the DEGs to the KEGG pathways database.

### 2.6. Statistical Analysis

SIGMA PLOT v 14.5 was used to perform all statistical analyses and create bar graphs. Each analysis was repeated three times in a completely randomized block design setup. Two-tailed Welch’s *t*-test of *p* < 0.05 was used to determine significance levels in each experiment and to compare the control and treated plants.

## 3. Results

### 3.1. Shoot Length and Total Biomass Increased in Plants Treated with Citrate and CTAB-AuNPs

**Figure 1:**
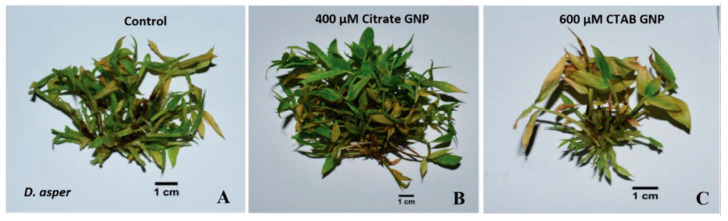
In vitro shoot proliferation and total biomass production in proliferated *D. asper* under control (MS + 2 mg·L^−1^ BAP) and AuNP treated plants after 30 days. Biomass proliferation in (**A**) control; (**B**) 400 µM citrate-AuNP treated; and (**C**) 600 µM CTAB-AuNP treated. Scale bar = 1 cm.

**Figure 2:**
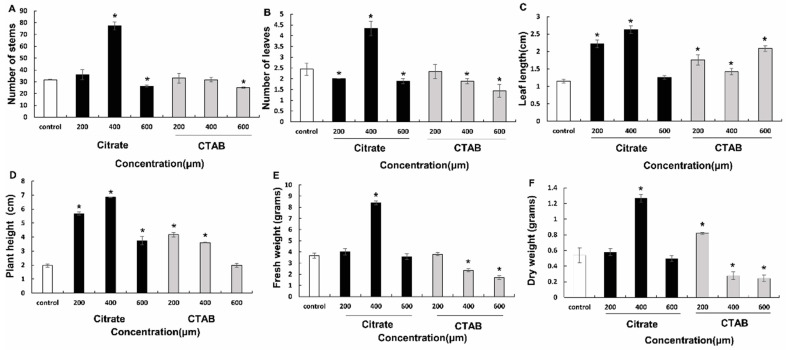
Effect of citrate and CTAB-AuNPs on growth and biomass enhancement of *D. asper*. (**A**) effect on number of stems; (**B**) effect on number of leaves; (**C**) effect on leaf length; (**D**) effect on plant height; (**E**) effect on fresh weight; and (**F**) effect on dry weight. The error bar represents standard error of three biological replicates. * Indicates significantly reduced and enhanced parameters in comparison to the control.

Both citrate- and CTAB-AuNPs considerably improved biomass proliferation in a concentration-dependent manner (Figure 1). Each concentration of citrate- and CTAB-AuNPs significantly induced plant height, with the maximum plant height observed in plants treated with 400 µM citrate-AuNPs (6.84 cm). The number of shoots in plants treated with citrate-AuNPs increased when the concentration was increased from 200 µM (36) to 400 µM (77.33); however, the number of shoots in plants treated with 600 µM CTAB-AuNPs (25) also had significantly lesser number of shoots than control plants (31.66). Plants treated with 600 µM CTAB-AuNPs (25) also had significantly lesser number of shoots than control plants. Increasing the concentration of citrate-AuNPs from 200 µM to 400 µM, significantly increased plant biomass (4.03 g to 8.4 g) compared to control (3.67 g). However, increasing CTAB-AuNPs concentration from 400 µM to 600 µM significantly reduced plant biomass from 2.35 g to 1.71 g (Figure 2).

### 3.2. Photosynthetic Pigment and Total Soluble Sugar Contents Increased after AuNPs-Treatment

**Figure 3:**
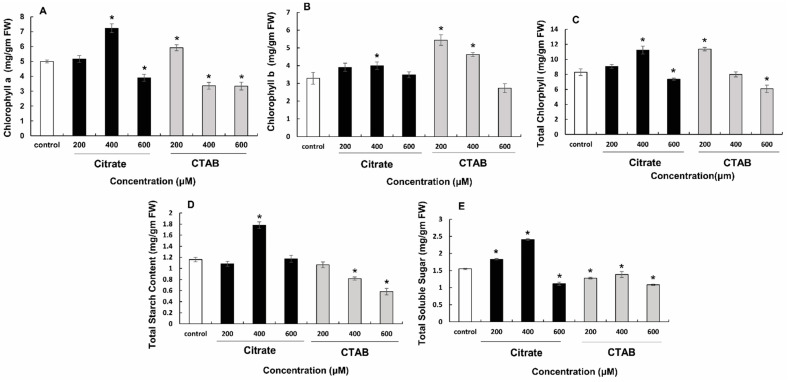
Effect of citrate and CTAB-AuNPs on photosynthetic pigments, sugar and starch content of *D. asper*. Effect on chlorophyll a (**A**), chlorophyll b (**B**) and total chlorophyll (**C**) content, respectively; (**D**) effect on total starch content; (**E**) effect on total soluble sugar content. The error bar represents the standard error of three biological replicates. * Indicates significantly reduced and enhanced concentrations in comparison to the control.

Citrate- and CTAB-AuNPs differentially affected total chlorophyll pigment and soluble sugar contents in a concentration-dependent manner. Total chlorophyll and chlorophyll a and chlorophyll b contents in plants treated with 600 µM citrate-AuNPs were significantly lower (3.89, 3.47, and 7.37 mg·g^−1^ fresh weight [FW], respectively) while those in plants treated with 400 µM citrate-AuNPs were significantly higher (7.23, 3.99, and 11.22 mg·g^−1^ FW, respectively) than in control plants (4.99, 3.29, and 8.29 mg·g^−1^ FW, respectively). Treatment with 200 µM CTAB-AuNPs also enhanced total chlorophyll and chlorophyll a and b contents (5.9, 5.44, and 11.35 mg·g^−1^ FW, respectively) significantly compared to those in controls (Figure 3A–C).

Total soluble sugar level was significantly affected by both citrate- and CTAB-AuNPs in a concentration-dependent manner. As the concentration increased from 200 µM (1.83 mg·g^−1^ FW) to 400 µM (2.40 mg·g^−1^ FW), soluble sugar content increased significantly. In contrast, treatment with 600 µM citrate-AuNPs significantly reduced soluble sugar content in plants (1.55 mg·g^−1^ FW). In plants treated with 600 µM CTAB-AuNPs, there were significant reductions in shoot number (25) and sugar content (1.084 mg·g^−1^ FW), as shown in Figure 3D,E.

### 3.3. Total Phenolics, MDA and SOD Activity Increased after AuNPs Treatment

**Figure 4:**
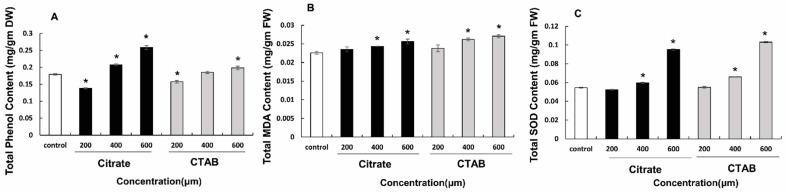
Effect of citrate and CTAB-AuNPs on antioxidant activity and lipid peroxidation of *D. asper*. (**A**) effect of total phenolic content; (**B**) effect of malondialdehyde content; and (**C**) effect on superoxide dismutase enzyme activity. The error bar represents standard error of three biological replicates. * Indicates significantly reduced and enhanced concentrations in comparison to the control.

Phenolic content increased significantly in plants treated with 400 µM and 600 µM citrate- and CTAB-AuNPs. Maximum phenolic content was observed in plants treated with 600 µM citrate-AuNPs (0.25 mg·g^−1^ DW). Similarly, SOD enzyme activity increased significantly in plants treated with citrate- and CTAB-AuNPs in a concentration-dependent manner. Plants treated with 600 µM CTAB-AuNPs (0.103 mg·g^−1^ FW) showed the highest SOD activity. Moreover, MDA content increased significantly in plants treated with citrate- and CTAB-AuNPs in a concentration-dependent manner. The highest MDA content was observed in plants treated with 600 µM CTAB-AuNPs (0.027 mg·g^−1^ FW), as shown in Figure 4.

### 3.4. Transcriptome Analysis

**Figure 5:**
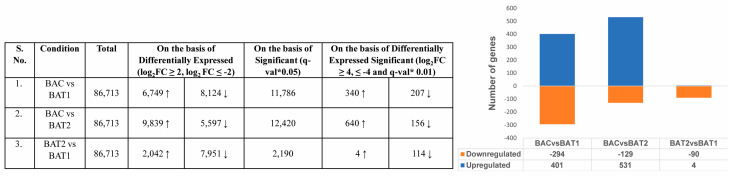
Differentially expressed genes in different treatment conditions in *D. asper*. * q-value is a corrected *p*-value # Upregulated Genes-↑, Downregulated Genes-↓. BAC represents control, BAT1 represents 400 µM citrate AuNPs and BAT2 represents 600 µM CTAB AuNPs.

To study the genes involved in biomass enhancement in *D. asper* due to nano-elicitation, RNA-seq analysis was performed on 30 days-old control and treated plants. Raw reads ranging from 3918968–26131133 produced by Illumina sequencing were analyzed (Supplementary Table S1). By filtering and eliminating adaptor sequences, high quality reads were obtained from raw reads using NGSQC toolkit v2.3; in total, 3670833–24406137 reads were obtained (Supplementary Table S2). The hits obtained for DEGs were annotated using UniProt via BLASTx (Supplementary Table S3; Figure 5).

### 3.5. Differentially Expressed Genes after Nano-Elicitation

**Figure 6:**
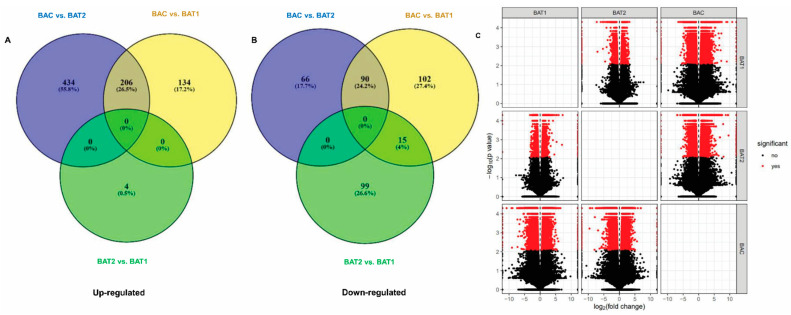
Venn diagram showing number of differentially expressed genes in different treatment conditions (400 µM citrate vs. Control, 600 µM CTAB vs. Control, 400 µM citrate vs. 600 µM CTAB) (**A**) Upregulated, (**B**) Downregulated genes. BAC represents control, BAT1 represents 400 µM citrate AuNPs and BAT2 represents 600 µM CTAB AuNPs. (**C**) Volcano plot showing the significant differentially expressed genes.

A total of 86,713 genes were identified; 401, 531 and 4 of these were significantly up-regulated in BAC vs. BAT1, BAC vs. BAT2 and BAT2 vs. BAT1 respectively, whereas 294, 129 and 90 were significantly down-regulated in BAC vs. BAT1, BAC vs. BAT2 and BAT2 vs. BAT1, respectively (Supplementary Tables S4 and S5). The Venn diagram representing significantly up-regulated and down-regulated genes between control and treated plants shows that 206 up-regulated and 90 down-regulated genes are common between BAC vs. BAT2 and BAC vs. BAT1, respectively (Figure 6). Similarly, 15 down-regulated genes were common between BAC vs. BAT1 and BAT2 vs. BAT1. Moreover, 66, 102 and 99 significantly down-regulated genes and 434, 134 and 4 significantly up-regulated genes were found in BAC vs. BAT2, BAC vs. BAT1 and BAT2 vs. BAT1, respectively. Additionally, the volcano plots show the distribution of significant DEGs (Figure 6A–C).

**Figure 7:**
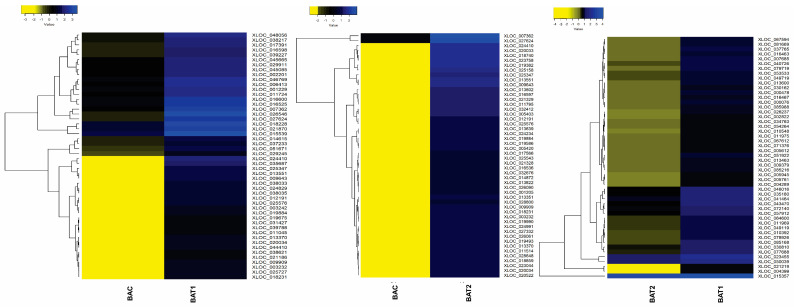
Heat maps showing differentially upregulated genes between control and AuNPs treated plants of *D. asper*. Heat map prepared at a log2fold scale of −4 to +4. BAC represents control; BAT1 represents 400 µM citrate AuNPs; and BAT2 represents 600 µM CTAB AuNPs.

**Figure 8:**
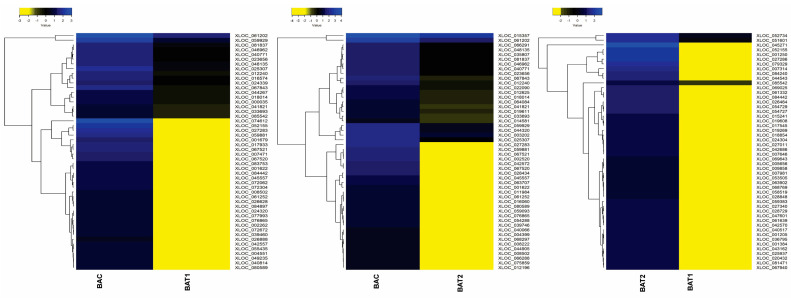
Differentially downregulated genes between control and AuNPs treated plants in *D. asper*. Heat map prepared at a log2fold scale of −4 to +4. BAC represents control; BAT1 represents 400 µM citrate AuNPs; and BAT2 represents 600 µM CTAB AuNPs.

**Figure 9:**
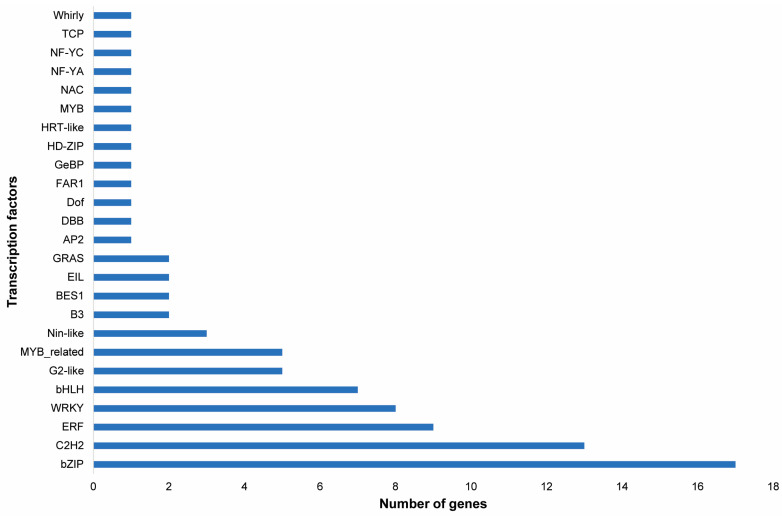
Differentially expressed transcription factors among control, citrate and CTAB coated gold nanoparticles.

Furthermore, the heatmap scaled on log2 fold change values shows the expression pattern of the top 50 differentially up-regulated and down-regulated genes. The heatmap showed the up-regulation of genes such as the auxin responsive protein IAA19 (XLOC_067594), clathrin assembly protein (XLOC_007685), histidine kinase 4 (XLOC_053533), SNARE 13-like (XLOC_016467), pyruvate kinase (XLOC_005945) and WAT1-related protein (XLOC_011969) in BAT2 vs. BAT1, indicating their role in growth regulation, cell wall formation and anti-oxidant potential. The down-regulated genes in BAT2 vs. BAT1 were NAM protein (XLOC_052155), glycosyl transferase (XLOC_015241), E3 ubiquitin protein ligase (XLOC_068769), MYB30 (XLOC_061639) and calcium sensing receptor (XLOC_040517). In BAC vs. BAT1, genes involved in cell wall synthesis and growth, such as cell wall protein gp-1-like (XLOC_006413), aldehyde dehydrogenase (XLOC_046769), phospholipase C (XLOC_037233) and glucose-6-phosphate translocator2 (XLOC_045665), were up-regulated. In contrast, the down-regulated genes were pathogenesis related protein (XLOC_016574), WRKY70 TF (XLOC_044267) and cytokinin oxidase (XLOC_025307). Because WRKY 70 is involved in defense signaling pathway and cytokinin oxidase catalyses the degradation of cytokinin phytohormone, BAT1-treated plants showed better growth. Furthermore, in BAC vs. BAT2, up-regulation of senescence and phytohormone degradation related genes, such as cytokinin oxidase/dehydrogenase (XLOC_007362), senescence specific cysteine protease (XLOC_0025347), F-box protein (XLOC_0242434), cysteine protease (XLOC_018231) and anthocyanin reductase (XLOC_023044), and down-regulation of aquaporin TIP4-1 (XLOC_066291), metal transporter NRAT1 (XLOC_040771), peroxidase P7-like (XLOC_012240) and oxalate_CoA ligase (XLOC_061202) were observed (Figure 7 and Figure 8). Besides these, 25 transcription factors were also differentially regulated, as shown in Figure 9.

### 3.6. Functional Annotation and GO Analysis of Growth Responsive DEGs

**Figure 10:**
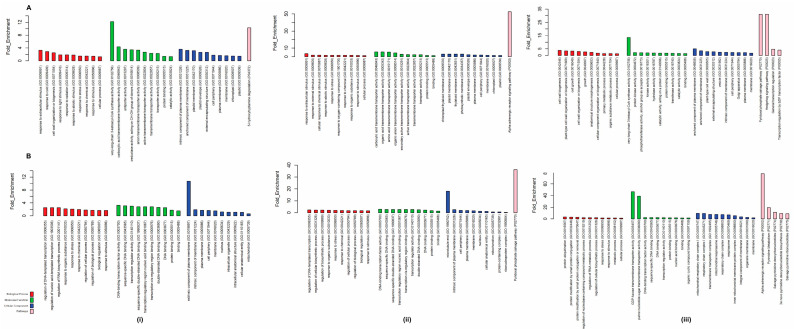
GO enrichment analysis for upregulated (**A**) and downregulated (**B**) genes. Biological processes, cellular components, molecular functions and pathway enrichment analysis in BAT1-BAC (**i**), BAT2-BAC (**ii**) and BAT1-BAT2 (**iii**).

GO analysis showed that growth responsive DEGs were enriched in molecular, cellular and biological processes. GO analysis was performed separately for up-regulated and down-regulated genes under each treatment condition, and significant annotation was represented as bar plots. The following biological functions were up-regulated in BAC vs. BAT1: extracellular stimulus, cold response, cell wall organization and response to light stimulus. The up-regulated biological processes in BAC vs. BAT2, were external and internal stimulus and abiotic stimulus, while those in BAT2 vs. BAT1 were cell wall biogenesis and cell growth. The down-regulated biological processes were transcription, RNA biosynthesis and response to organic substances in BAC vs. BAT1; transcription and response to organic substances in BAC vs. BAT2; and protein ubiquitination and protein conjugation in BAT2 vs. BAT1. The up-regulated molecular functions were 3-ketoacyl-CoA synthase activity, transporter activity and antiporter activity in BAC vs. BAT1; carboxylic acid, organic acid and amino acid transporter activities in BAC vs. BAT2; and 3-ketoacyl-CoA synthase activity and protein kinase activity in BAT2 vs. BAT1. The down-regulated molecular functions were transcription factor activity and protein binding activity in BAC vs. BAT1 and BAC vs. BAT2, respectively, and GDP-fructose transmembrane transporter activity and transcription factor activity in BAT2 vs. BAT1. The up-regulated cellular components in BAC vs. BAT1 and BAC vs. BAT2 were plasma membrane, chloroplast and plastid, whereas those in BAT2 vs. BAT1 were cell wall, vesicle and Golgi apparatus 3-ketoacyl-CoA synthase activity and protein kinase activity. In contrast, the down-regulated cellular functions in BAC vs. BAT1, BAC vs. BAT2 and BAT2 vs. BAT1 were plasma membrane, nucleus and mitochondrion (Figure 10).

### 3.7. Analysis of Metabolic Pathways Triggered by AuNP Treatments

**Figure 11:**
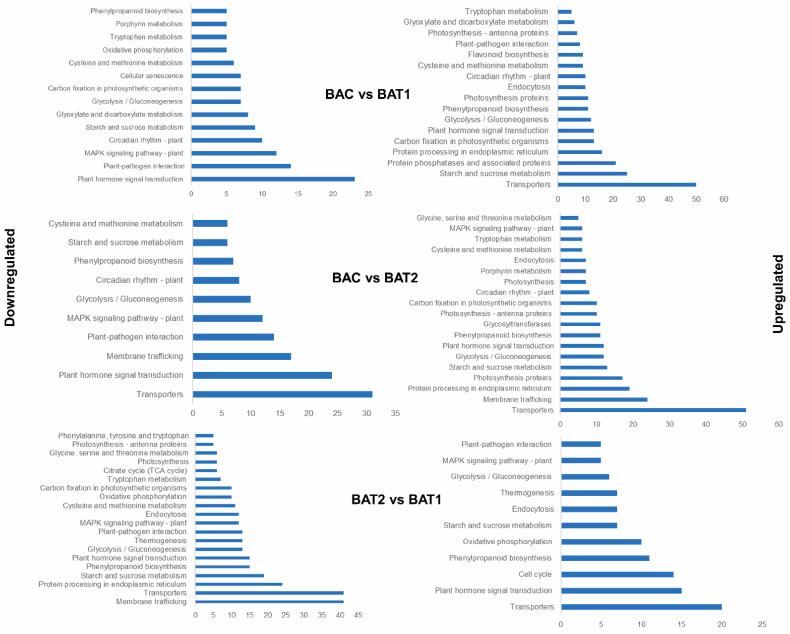
KEGG pathway analysis. KEGG enriched plant hormone signal transduction, transport, plant pathogen interaction and photosynthesis in BAC vs. BAT1, BAC vs. BAT2 and BAT2 vs. BAT1.

Using KEGG with Arabidopsis as reference (Supplementary Table S6), the significantly up- and down-regulated genes were analyzed for metabolic pathways. KEGG pathways with a gene number ≥ 5 were considered. KEGG analysis showed that 14, 10 and 20 pathways were significantly down-regulated, whereas, 17, 19 and 11 pathways were significantly up-regulated in BAC vs. BAT1, BAC vs. BAT2 and BAT2 vs. BAT1, respectively. The main up-regulated pathways in BAC vs. BAT1 were transporters (50), starch and sucrose metabolism (25), protein phosphatases and associated proteins (21), plant hormone signal transduction (13), photosynthesis proteins (11), endocytosis (10) and circadian rhythm (10). In BAC vs. BAT2, the significantly up-regulated pathways were transporters (51), membrane trafficking (24), photosynthesis proteins (17), starch and sucrose metabolism (13), plant hormone signal transduction (12), phenylpropanoid biosynthesis (11) and glycosyltransferases (11). The significantly enriched pathways in BAT2 vs. BAT1 were plant hormone signal transduction (15), cell cycle (14), phenylpropanoid biosynthesis (11), oxidative phosphorylation (10), starch and sucrose metabolism (7), endocytosis (7) and MAPK signaling pathway. The down-regulated pathways in BAC vs. BAT1 included plant hormone signal transduction (23), plant pathogen interaction (14), MAPK signaling pathway (12), cellular senescence (7) and glyoxalase and decarboxylase metabolism (7). Comparatively down-regulated pathways in BAC vs. BAT2 included sucrose metabolism (6), phenylpropanoid biosynthesis (7) and membrane trafficking (13). In BAT2 vs. BAT1, down-regulated genes were associated with photosynthetic proteins (5), endocytosis (12), MAPK pathway (12), membrane trafficking (41) and transporters (41) (Figure 11).

## 4. Discussion

The use of NPs in agronomy and biotechnology for mass scale propagation and sec-ondary metabolite production in different plants has increased significantly. Therefore, it is crucial to investigate how plants are affected by metallic and non-metallic NPs. AuNPs were previously found to be hazardous at concentrations ≥100 mg·L^−1^ and particle diame-ter <5 nm; however, their basic mechanism remains unclear. Under natural conditions, the chemistry of the coated material on NPs can change, altering the characteristics and stability of nanomaterials; however, surface alterations may stabilize them [15]. The growth of a species may be greatly influenced by the size and concentration of NPs. Surface coating is crucial for the stabilization of NPs, despite the risk of their aggregation in nutrient media. Positively or negatively charged coatings can affect how NPs interact with plasma membrane. Positively charged NPs assemble more readily on the cell surface than negatively charged ones because of electrostatic attraction. In contrast, negatively charged NPs can pass through the plasma membrane by forming potent covalent bonds [2]. The out-comes of the present study suggest that electrostatic repulsion may be overcome by smaller particles, regardless of charge, and that the difference in the metal ion gradient between, within and outside the cell may be a critical factor in internalization of NPs.

In this study, citrate-AuNPs and CTAB-AuNPs significantly boosted shoot prolifera-tion, total biomass production, photosynthetic pigment content and anti-oxidant potential in bamboo in a dose-dependent manner [15]. Our study revealed that AuNPs boosted all growth parameters at optimum concentration but caused deterioration at higher concen-trations. Despite being active, NPs at lower concentrations are not translocated to roots and shoots [24]. Previous studies showed that AgNPs cannot penetrate the roots at lower concentrations [25,26].

According to Acharya et al., 2019 [27], treatment with AuNPs boosted the overall light absorption by chlorophyll, accelerating the photochemical reaction and increasing the reducing power (NADPH^+^) and energy (ATP) available for CO_2_ fixation. The sugar content in treated plants approximately doubled, indicating better CO_2_ fixation [28]. Our study demonstrated that higher chlorophyll content increases CO_2_ fixation, increasing biomass produced per plant. Chlorophyll content is used to gauge a plant’s capacity for photosynthetic activity affecting growth and development. Carotenoids absorb extra light and protect against photo-oxidation, which is harmful to the chlorophyll molecules [15]. In the present study, treatment with 400 µM citrate-AuNPs and 200 µM CTAB-AuNPs increased photosynthetic pigments. The up-regulation of reactive oxygen species (ROS)-scavenging enzymes may underlie this improvement in chlorophyll content in AuNPs-treated plants [29]. Total soluble sugars are essential for plant development, metabolism and maintenance of ROS balance in cells [30]. In our study, plants treated with 400 µM citrate-AuNPs and 200 µM CTAB-AuNPs had higher levels of total soluble sugar, indicating that the NPs positively influenced cellular metabolism, resulting in enhanced plant growth. Bananas grown in vitro have been shown to contain more chlorophyll and total soluble sugar when exposed to Ca-AuNPs and Ca-AuNP nano-composites coated with polyethylene glycol methacrylate [31].

MDA acts as a marker for oxidative stress, and plantlets supplemented with 400 µM AuNPs showed accelerated growth and decreased MDA and proline contents due to en-hanced anti-oxidant potential of enzymes. Plant biomass decreases at higher concentra-tions of AuNPs (600 µM) due to increased free radical formation, suggesting oxidative stress [32]. ROS are also involved in secondary metabolic pathways. The MDA level in-creased steadily with increase in citrate-AuNPs and CTAB-AuNPs concentrations. Never-theless, the treated plantlets consistently accumulated lower MDA concentrations than the control plants, regardless of the AuNPs concentration. This clearly demonstrates that AuNPs may increase the effectiveness of electron exchange/transport reactions, thus re-ducing the oxidative load [33]. Phenolics have redox characteristics that enhance anti-oxidant potential of plants. By neutralizing the ROS produced during electron transport chain cycles, phenolic substances protect cells from oxidation [34]. The AuNPs used in this study increased total phenolic content in a concentration-dependent manner. NPs promote the generation of phenolics, increasing systemic acquired resistance [35].

The developmental switch regulating biomass/growth differences between control and treated plants is highly regulated by internal cues [36]. These regulatory components and mechanisms involved in sudden changes in the plant are not well-known. Thus, transcriptome analysis of control and treated *D. asper* micro-plantlets was performed to identify the genetic machinery regulating the visible changes in growth. In our transcriptome analysis, the filtered reads led to higher gene coverage, and high throughput sequencing using GO and KEGG showed the dominance of ‘biological and metabolic processes, growth and stress related GO terms’ in the biological category and ‘plant hormone signal transduction, secondary metabolite and amino acid biosynthesis’ in KEGG analysis are differentially regulated under control and treated conditions. This study identified growth responsive DEGs/transcripts involved in several biological, cellular and molecular processes.

The current study identified several transporters, including the V-type H^+^-transporting ATPase subunit F, MFS transporter, ABC transporter 1, VGAT, Nrat1 and P-type Ca^2+^ transporter type 2C. These transporters handle various substances. The plasma membrane-localized Nrat1 has a broad substrate range including Fe^2+^, Zn^2+^, Mn^2+^, Co^2+^, Cd^2+^, Cu^2+^, Ni^2+^ and Pb^2+^. NRAMP proteins are conserved and vital for metal ion balance. For instance, *AtNRAMP3* and *AtNRAMP4* in Arabidopsis transport iron from vacuoles to support plant growth [37]. Phospholipase C splits phosphatidylinositol 4,5-bisphosphate into DAG and IP3, acting as secondary messengers for cellular processes and other signaling molecule synthesis [38]. Similarly, the expression of clathrin assembly proteins increases when exposed to 400 µM citrate, indicating increased metal ion intake. WAT1-related proteins, involved in secondary cell wall synthesis, also enhance in similar treatment. Our study confirms that anti-oxidant capacity and biomass accumulation are enhanced in plantlets treated with 400 µM citrate.

This study revealed several significant DEGs that may be involved in mediating nano-elicited growth and anti-oxidant behavior of plants, including metal transporter *NRAT1*, cytokinin oxidase/dehydrogenase *CKX3* gene, PR-5, aquaporin TIP4-1, WRKY TF 70, peroxidase P7-like, NAC2 TF, aldehyde de-carboxylase, cell wall protein gp-1-like, isoamylase, phospholipase C, ferric reduction oxidase 7, glycosyltransferase, ABC transporter 1, E3 ubiquitin protein ligase, stromal cell-derived factor-2 like protein, IAA 19, SNARE-13-like, WAT1-related protein, ferritin-1, anthocyanin reductase and various transcription factors. These DEGs were involved in metal uptake, protein ubiquitination, cell wall synthesis, redox homeostasis, ethylene activated signaling and WRKY-regulated photomorphogenesis. AuNPs enter plantlets via basal shoot regions and are translocated by various metal transporters (NRAT1 XLOC_040771), leading to modulation of photo-morphogenetic behavior and resulting in enhanced growth and anti-oxidant potential [39]. BAT1, i.e., 400 µM citrate AuNPs, showed best biomass growth and enhanced anti-oxidant potential of bamboo. Cytokinin oxygenase/dehydrogenase (XLOC_007362), isoamylase2 (XLOC_014615) and phospholipase-C (XLOC_037233) were up-regulated and may be linked to enhanced anti-oxidant activities. Moreover, MYBAS2 (XLOC_029911), cell wall protein gp-1-like (XLOC_006413) and proteins of photosystem 2 (XLOC_002201), which were up-regulated, are involved in photosynthesis and growth processes. Thus, plantlets treated with 400 µM citrate-AuNPs had better overall growth.

Transcription factors have a major role in modulating signaling pathways [40]. We identified differential expression of transcripts encoding *MYB*, *WRKY*, *DOF*, *GRAS*, *C2H2*, *TCP*, *B3*, *ERF*, *NAC*, *BES1* and *bZIP* TFs during growth and stress responses. These TFs are involved in the regulation of stomata opening and closing, anti-oxidant potential, cu-ticular wax biosynthesis, membrane modulation and cell wall biosynthesis [41]. Therefore, functional analysis of these genes to reveal their roles in modulating growth and oxidative behavior in bamboo might identify novel candidates to develop more resilient varieties.

## 5. Conclusions

The current study, for the first time, demonstrated that MS supplemented with BAP (2 mg·L^−1^) and 400 µM citrate-AuNPs concentration increases anti-oxidant properties of bamboo species while also promoting shoot growth in a concentration-dependent manner. Citrate-AuNPs and CTAB-AuNPs enhanced anti-oxidant potential and synthesis of phenolic derivatives, thus reducing ROS production. Additionally, charged AuNPs can be used in tissue culture for quick and effective plant propagation as well as to reduce ROS production in bamboo species. Transcriptome analysis of in vitro regenerated shoots of control and treated plants has shown ways to find key regulators of growth changes in *D. asper*. It revealed various molecular and biological pathways modulated by various plant hormones and growth responsive genes after NP-treatment. Overall, our study provided significant insights into the transcriptome dynamics of nano-elicitation in bamboo. This study not only advances bamboo species propagation and applications but also basic research in genetic engineering and stress response.

## Data Availability

The data are contained within the article and supplementary materials.

## Supplementary Materials

The following supporting information can be downloaded at: https://www.mdpi.com/article/10.3390/genes14091725/s1, Supplementary Table S1. Sequencing Data Report (Raw data); Supplementary Table S2. Sequencing Data Report (High Quality trimmed data); Supplementary Table S3. Blast X data; Supplementary Table S4. Raw data of significant differentially downregulated genes in BAC vs. BAT1, BAC vs. BAT2, respectively; Supplementary Table S5. Raw data of differentially downregulated genes in BAT2 vs. BAT1; Supplementary Table S6. Identification of genes regulating potential routes/pathways using KEGG database.
