# Supplementary material for: Physio-Biochemical Integrators and Transcriptome Analysis Reveal Nano-Elicitation Associated Response during Dendrocalamus asper (Schult. and Schult. F.) Backer ex K. Heyne Micropropagation
Source: Genes (Basel). 2023 Aug 29;14(9):1725. doi: 10.3390/genes14091725 (PMC10530697; doi:10.3390/genes14091725)
Supplement: Supplementary file 1 [file genes-14-01725-s001.zip › Supplementary Table S1.pdf]

SupplementaryTable S1: Sequencing Data Report (Raw data)

S. No.	Sample Name	No. of Reads	Sequence_Length	%GC
1.	AN1_S4_R1.fastq	18989168	35-149	53
2.	AN1_S4_R2.fastq	18989168	35-149	51
3.	AN2_S10_R1.fastq	8571000	35-149	52
4.	AN2_S10_R2.fastq	8571000	35-149	50
5.	AN3_S16_R1.fastq	26131133	35-149	53
6.	AN3_S16_R2.fastq	26131133	35-149	50
7.	AN4_S22_R1.fastq	8883367	35-149	53
8.	AN4_S22_R2.fastq	8883367	35-149	50
9.	AN5_S27_R1.fastq	3918968	35-149	53
10.	AN5_S27_R2.fastq	3918968	35-149	50
11.	AN6_S33_R1.fastq	11685059	35-149	51
12.	AN6_S33_R2.fastq	11685059	35-149	48
13	AN7_S39_R1.fastq	15234475	35-149	56
14.	AN7_S39_R2.fastq	15234475	35-149	53
15.	AN8_S50_R1.fastq	17584435	35-149	53
16.	AN8_S50_R2.fastq	17584435	35-149	51
17.	AN9_S5_R1.fastq	16307257	35-149	54
18.	AN9_S5_R2.fastq	16307257	35-149	52
