# Supplementary material for: Physio-Biochemical Integrators and Transcriptome Analysis Reveal Nano-Elicitation Associated Response during Dendrocalamus asper (Schult. and Schult. F.) Backer ex K. Heyne Micropropagation
Source: Genes (Basel). 2023 Aug 29;14(9):1725. doi: 10.3390/genes14091725 (PMC10530697; doi:10.3390/genes14091725)
Supplement: Supplementary file 1 [file genes-14-01725-s001.zip › Supplementary Table S2.pdf]

Supplementary Table S2: Sequencing Data Report (High Quality trimmed data)

S. No.	Sample Name	No. of Reads	Sequence_Length	%GC
1.	AN1_S4_R1.fastq	17701746	54-134	54
2.	AN1_S4_R2.fastq	17701746	54-134	49
3.	AN2_S10_R1.fastq	8009089	54-134	53
4.	AN2_S10_R2.fastq	8009089	54-134	48
5.	AN3_S16_R1.fastq	24406137	54-134	53
6.	AN3_S16_R2.fastq	24406137	54-134	49
7.	AN4_S22_R1.fastq	7520155	54-134	53
8.	AN4_S22_R2.fastq	7520155	54-134	48
9.	AN5_S27_R1.fastq	3670833	54-134	54
10.	AN5_S27_R2.fastq	3670833	54-134	49
11.	AN6_S33_R1.fastq	11211587	54-134	50
12.	AN6_S33_R2.fastq	11211587	54-134	47
13	AN7_S39_R1.fastq	14246009	54-134	56
14.	AN7_S39_R2.fastq	14246009	54-134	52
15.	AN8_S50_R1.fastq	16518686	54-134	54
16.	AN8_S50_R2.fastq	16518686	54-134	50
17.	AN9_S5_R1.fastq	14838377	54-134	54
18.	AN9_S5_R2.fastq	14838377	54-134	50
